# Identification of a prognostic 28-gene expression signature for gastric cancer with lymphatic metastasis

**DOI:** 10.1042/BSR20182179

**Published:** 2019-05-02

**Authors:** Chao Zhang, Li-wei Jing, Zhi-ting Li, Zi-wei Chang, Hui Liu, Qiu-meng Zhang, Qing-yu Zhang

**Affiliations:** 1 Department of Gastroenterology, Tianjin Medical University General Hospital, Tianjin 300052, China; 2 Department of Gastroenterology, Affiliated Hospital of North China University of Science and Technology, Tangshan, Hebei 063000, China; 3 National Institute of Development and Strategy, Renmin University of China, Beijing 100872, China

**Keywords:** COX, Elastic net, gastric cancer, lymph node metastasis, prognosis, VEGF-C

## Abstract

Gastric cancer (GC) patients have high mortality due to late-stage diagnosis, which is closely associated with lymph node metastasis. Exploring the molecular mechanisms of lymphatic metastasis may inform the research into early diagnostics of GC. In the present study, we obtained RNA-Seq data from The Cancer Genome Altas and used Limma package to identify differentially expressed genes (DEGs) between lymphatic metastases and non-lymphatic metastases in GC tissues. Then, we used an elastic net-regularized COX proportional hazard model for gene selection from the DEGs and constructed a regression model composed of 28-gene signatures. Furthermore, we assessed the prognostic performance of the 28-gene signature by analyzing the receive operating characteristic curves. In addition, we selected the gene PELI2 amongst 28 genes and assessed the roles of this gene in GC cells. The good prognostic performance of the 28-gene signature was confirmed in the testing set, which was also validated by GSE66229 dataset. In addition, the biological experiments showed that PELI2 could promote the growth and metastasis of GC cells by regulating vascular endothelial growth factor C. Our study indicates that the identified 28-gene signature could be considered as a sensitive predictive tool for lymphatic metastasis in GC.

## Introduction

Gastric cancer (GC) remains one of the leading causes of cancer mortality worldwide [1] despite the steady decline in its incidence and mortality over the past decades. Lower incidence of *Helicobacter pylori*, adoption of healthier diets and considerable advances in therapies for GC are the main contributors to this decline [2]. GC patients diagnosed at an early stage of disease have the 5-year survival rate of over 90% [3]. However, the majority of GC patients are diagnosed at an advanced stage, and the 5-year rate of overall survival is only 30% [1,4]. The high mortality rate in GC is mostly influenced by formation of lymph node metastases [5]. As the presence of lymphatic metastases has been reported as one of the most important prognostic predictors in GC, early identification of lymphatic metastases is critically important for the patient outcome [6]. Although the role of lymphatic metastases in GC has been intensely studied in recent years, the molecule mechanisms of their formation have not been completely understood [7]. Thus, exploring these mechanisms may help to find better methods of early diagnostics of GC.

The development of modern bioinformatics and next-generation sequencing has offered multiple efficient tools to analyze the molecular mechanisms of carcinogenesis at genetic level [8]. To date, plenty of effective diagnostic and prognostic cancer biomarkers are identified using the high-throughput screening methods [9]. For instance, Li and co-authors used the high-throughput screening method to identify the significantly different hub genes (CASR, CXCL12, and SST) that have the prognostic potential for GC treatment [10]. Recent studies have indicated that integrating multiple biomarkers into a single model have a relatively higher predictive accuracy as compared with single biomarkers [11,12]. Thereby, the Cox’s proportional hazards model regularized by elastic net method was utilized to select genes and construct gene signature regression models for predicting the incidence of various diseases [13,14].

In the present study, we used RNA-Seq data from The Cancer Genome Altas (TCGA) to identify the differentially expressed genes (DEGs) between lymphatic metastases and non-lymphatic metastases in GC tissues. Then we used the elastic net-regularized COX proportional hazard model to select the gene signature from the DEGs and construct a prediction model of lymphatic metastases. This way, we obtained a 28-gene signature for GC with lymphatic metastases. As the gene PELI2 had the most significant difference in expression and the highest accuracy in predicting the lymphatic metastases amongst 28 DEGs, the role of this gene in GC was further investigated.

## Materials and methods

### Microarray data and DEGs analysis

The level 3 RNAseq datasets (RNAseqV2 RSEM) of GC patients were obtained from TCGA (http://cancergenome.nih.gov/). The dataset included a total of 415 samples containing 48 lymphatic metastasis GC tissues and 367 non-lymphatic metastasis GC tissues. These are publicly available open-access data. Thus, no approval by a local ethics committee was needed.

The downloaded raw data from TCGA were corrected for background, normalized, and expression was calculated using the Bioconductor package (version 3.6) in R language (version 3.4.3). After preprocessing of raw data, the DEGs between lymphatic metastases and non-lymphatic metastases in the GC tissues were assessed using Limma package in Bioconductor (version 3.6). The DEGs were screened using the cut-off criteria, which were set as an absolute log 2-fold change (FC) >0.1 and an adjusted *P* value of< 0.05. Finally, a total of 212 DEGs were obtained.

### Elastic net-regularized COX proportional hazard model for gene signature identification

A total of 415 patient tissues were randomly divided into a training set and a testing set in 2:1 ratio. In the training set, an elastic net-regularized COX proportional hazard model was performed to select the variables as gene signature from the DEGs using the R package ‘glmnet’ (version 2.0.2). In addition, a lambda penalty for this regression model was optimized by 10-fold cross validation. Finally, a regression model composed of 28-gene signature was obtained.

To determine whether these 28 DEGs overlapped or correlated with each other, we evaluated the correlation amongst the DEGs based on the mean absolute Pearson’s correlation >0.6.

### Evaluation of the prognostic performance of the 28-gene signature

The receive operating characteristic (ROC) curve was used to assess the prognostic performance of the regression model composed of 28-gene signature. Moreover, the regression model was validated by the testing set and GSE66229 dataset, respectively. The GSE66229 dataset containing GC tissues with or without lymphatic metastases was downloaded from the Gene Expression Omnibus database (GEO) (http://www.ncbi.nlm.nih.gov/gds).

### Cell culture and transfection

Normal human gastric epithelial cell line (GES-1), well-differentiated gastric adenocarcinoma cancer cell line MNK-7, and undifferentiated GC cell line HGC-27 were provided by Shanghai Institute of Cell Biology, Chinese Academy of Sciences (Shanghai, China). Cells were cultured in DMEM supplemented with 10% FBS, 100 U/ml penicillin, and 0.1 mg/ml streptomycin at 37°C in an incubator with 5% CO_2_.

siRNAs for PELI2 (siPELI2), siRNA control, PELI2 expression plasmid, and empty plasmid were purchased from GenePharma CO., Ltd. (Shanghai, China). After culturing to 60% confluence, the cells were transfected with siPELI2, siRNA control, PELI2 plasmid or empty plasmid using Lipofectamine 3000 (Invitrogen, Carlsbad, CA, U.S.A.) according to the manufacturer’s instructions. After 48 h, the transfected cells were harvested for the consequent experiments.

### Quantitative real-time PCR

Total RNA from cells was extracted using TRIzol (Invitrogen; Thermo Fisher Scientifc, Inc.). For the reverse transcription, cDNA were generated by a Primescript™ RT reagent Kit (Takara, Dalian, China). qRT-PCR was carried out using SYBR Green PCR Master Mix (Takara) and monitored using an ABI 7900 sequence detection system (Applied Biosystems, CA, U.S.A.). The amplification steps were as follows: 95°C for 5 min, followed by 40 cycles of 95°C for 30 s, 60°C for 40 s, and 72°C for 1 min. β-Actin was used as an internal control. The mRNA expression was calculated by the 2^−ΔΔ*C*^_T_ method. The following primer sequences were used: PELI2: forward, 5′-CGC GCG CGG ATT TGA CTC TT-3′, reverse, 5′-CTG GGT GAA GCC CCC TCG TG-3′; β-actin: forward, 5′-TGA CGG GGT CAC CCA CAC TGT GCC CAT CTA-3′, reverse, 5′-CTA GAA GCA TTT GCG GTG GAC GAT GGA GGG-3′.

### Cell counting kit-8 assay

After transfection, HGC-27 cells were harvested, seeded at a concentration of 1000 cells per well into 96-well plates, and cultured for 24, 48, 72, and 96 h, respectively. At every time point, 10 μl CCK-8 solution was added to each well. The cells were then incubated for 1.5 h at 37°C. The absorbance of the samples at 450 nm was measured by a microplate reader.

### Colony formation assay

The transfected cells were trypsinized, washed with PBS, and seeded onto six-well plates. The cells were incubated with complete medium for 2 weeks with replacement of the medium every 3 days. Finally, the cells were fixed with 4% paraformaldehyde, stained with 0.1% crystal violet, and photographed.

### Transwell assay

The 24-well plates with 8 μm pore transwell chambers were used to assess the migration and invasiveness of the cells. After transfection, cells were digested with trypsin, washed with PBS, centrifuged, and suspended in a serum-free medium. For invasion assay, the cells (1 × 10^3^ cells/μl) were added into the upper chamber coated with Matrigel (BD Biosciences, Billerica, M.A, U.S.A.). For migration assay, the cells were seeded into the upper chamber without Matrigel.

The lower chamber in the above two assays was filled with medium containing 10% FBS. After 24 h of incubation at 37°C, the cells that invaded or migrated through the membrane were collected, fixed in 1% paraformaldehyde, and stained with crystal violet. Then the samples were inspected using an optical microscope (Olympus BX51 microscope, Olympus Corp., Tokyo, Japan) at 200× magnification.

### Western blot

Total protein from the cells was extracted using RIPA buffer, and its concentration was assessed by Bicinchoninic acid (BCA) methods. The protein samples were separated by 10% SDS-PAGE and transferred to a PVDF membrane. The membranes were blocked with 5% non-fat milk for 1 h and then incubated with primary antibodies against PELI2, vascular endothelial growth factor C (VEGF-C), and actin overnight. After rinsing with TBST, the membranes were incubated with the secondary antibodies at room temperature for 1 h. The membranes were then washed with blocking solution and imaged using ECL detection system. The gray level of target bands was analyzed and calculated by Quantity One software. β-Actin was used as an internal control.

### Tube formation assay

HGC-27 cells were transfected with siPELI2, siRNA control, PELI2 plasmid or empty plasmid, incubated to 50% confluence, and the complete medium was changed into the serum-free one.

After the cells were incubated for additional 48 h, the supernatant of the culture medium was collected, centrifuged at 1500 ***g*** for 5 min to remove dead cells, sterilized by filtration through 0.22 μm filter, and harvested as a conditioned medium.

Human lymphatic endothelial cells (HLECs) were collected, suspended in the conditioned medium, and seeded at a concentration of 2 × 10^4^ cells/well onto 96-well plates pre-coated with 40 μl Matrigel (BD Biosciences). After 16 h of incubation at 37°C, the tube structures were recorded by an inverted phase-contrast microscope (Eclipse Ti-S; Nikon Corporation, Tokyo, Japan) at 100× magnification.

### Statistical analyses

Statistical analysis was performed by SPSS software (version 16.0, SPSS, Inc., Chicago, IL, U.S.A.). The data were presented as the means ± S.D. Differences between two groups were analyzed by Student’s *t*test. In addition, one-way ANOVA followed by Student–Newman–Keuls *post hoc* test was used to analyze the data in more than two groups. A value of p <0.05 was considered as a statistically significant difference.

## Results

### DEGs in lymphatic and non-lymphatic metastases of GC

**Figure 1:**
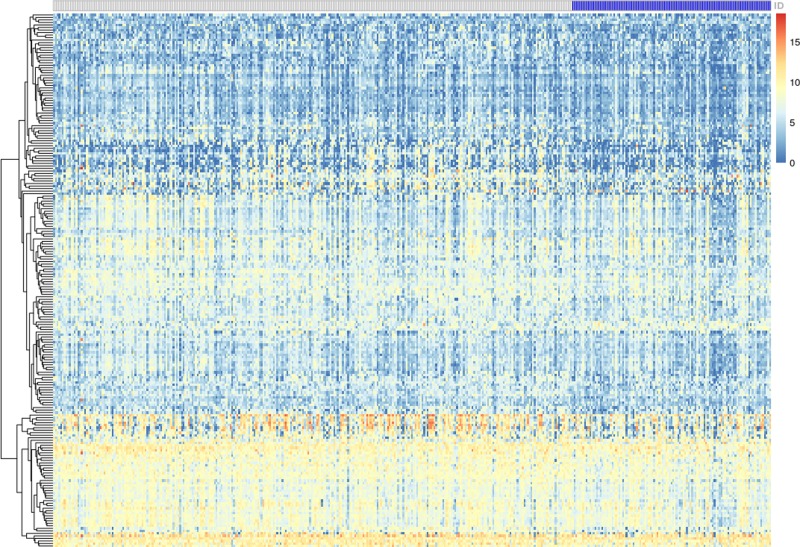
Heatmap for DEGs between lymphatic metastases and non-lymphatic metastases in GC The white lattice represents GC without lymph node metastases; the blue lattice represents GC with lymph node metastases.

The publicly available data on mRNA in GC patients were obtained from TCGA database and included 48 lymphatic metastasis GC tissues and 367 non-lymphatic metastasis GC tissues. After preprocessing of raw data, the data were analyzed by Limma package to identify the DEGs between lymphatic metastases and non-lymphatic metastases in GC tissues. Based on *P*<0.05 and |logFC| >0.1 cutoffs, we got a total of 212 DEGs (Figure 1) and listed the top 19 DEGs in Table 1. Amongst all genes, the expression of PELI2 showed the most significant difference between lymphatic and non-lymphatic metastases.

**Table 1:** The top nineteen of DEGs

	LogFC	Average Expression Level	t	*P* value	Adjusted *P* value	B
PELI2	−0.75081	8.570977	−5.77457	1.52E-08	0.000311	8.609372
HDC	−0.86562	4.197	−4.64017	4.68E-06	0.03758	3.670639
EFNA5	−0.83936	7.034063	−4.60485	5.50E-06	0.03758	3.532061
EMB	−0.61859	9.866523	−4.30995	2.04E-05	0.060415	2.411944
DYNC2H1	−0.71428	6.633374	−4.27667	2.36E-05	0.060415	2.289714
CPA3	−0.91607	7.456398	−4.13684	4.27E-05	0.097172	1.785648
CARD11	−0.78522	8.568952	−4.09275	5.13E-05	0.10508	1.629879
IL1RL1	−0.84825	4.529932	−4.06467	5.76E-05	0.106297	1.531462
PLEKHH2	−0.6827	7.472315	−4.02473	6.78E-05	0.106297	1.392571
MEIS3P1	−0.55995	7.589402	−4.01642	7.02E-05	0.106297	1.363813
C6orf141	−0.80589	5.702983	−3.94956	9.20E-05	0.106297	1.134534
IRAK3	−0.62369	8.362277	−3.94168	9.50E-05	0.106297	1.107759
PRKAR2B	−0.63456	7.601511	−3.93178	9.88E-05	0.106297	1.074148
SIGLEC6	−0.80939	3.706042	−3.91974	0.000104	0.106297	1.033407
ZBTB10	−0.58604	7.968481	−3.85035	0.000137	0.114459	0.800859
ABI3BP	−0.91925	8.320198	−3.82915	0.000148	0.114459	0.730571
TYRP1	−0.93929	4.833045	−3.79937	0.000167	0.114459	0.632429
ENPP5	−0.83653	5.863931	−3.79072	0.000173	0.114459	0.604049
ARL14	−0.86901	7.552498	−3.72769	0.00022	0.114459	0.399152

### The elastic net-regularized COX proportional hazard model for gene signature identification

**Figure 2:**
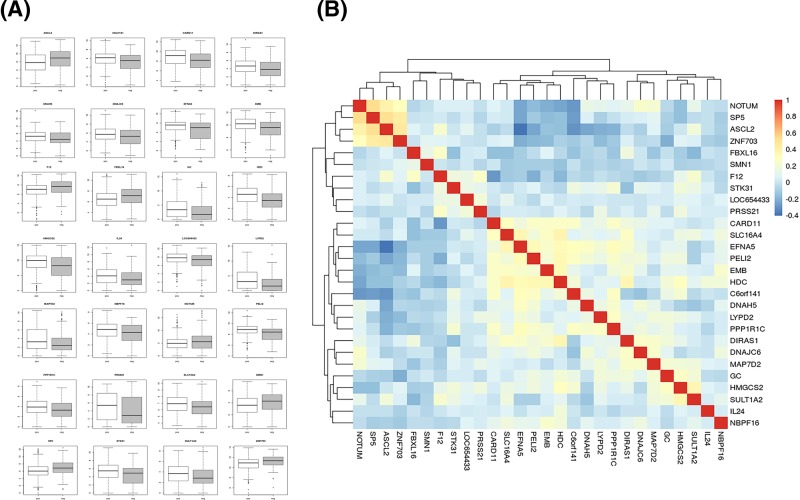
The 28-gene signature constructed by an elastic net-regularized COX proportional hazard model (**A**) The total 28 DEGs expression levels in GC patients with or without lymphatic metastases. (**B**) The correlation amongst the 28 DEGs was listed. ‘pos’ represents GC patients with lymphatic metastases; ‘neg’ represents GC patients without lymphatic metastases.

The elastic net-regularized COX proportional hazard model in the training set was performed to select the gene signature from the DEGs and to construct a prediction model of lymphatic metastasis using R package ‘glmnet’. The prediction model identified a 28-gene signature: ASCL2, C6orf141, CARD11, DIRAS1, DNAH5, DNAJC6, EFNA5, EMB, F12, FBXL16, GC, HDC, HMGCS2, IL24, LOC654433, LYPD2, MAP7D2, NBPF16, NOTUM, PELI2, PPP1R1C, PRSS21, SLC16A4, SMN1, SP5, STK31, SULT1A2, and ZNF703. The expression of the signature gene in GC with or without lymphatic metastases is shown in Figure 2A. There was no high correlation amongst these 28 DEGs, which further successfully validated 28-gene signature (Figure 2B).

### Evaluation of the prognostic performance for the regression model

To evaluate the prognostic performance of the regression model composed of 28-gene signature, we analyzed the ROC curve and calculated the area under the ROC curve (AUC) for the model in the testing set and GSE66229 dataset. The higher AUC reflects a better prognostic performance of the model.

**Figure 3:**
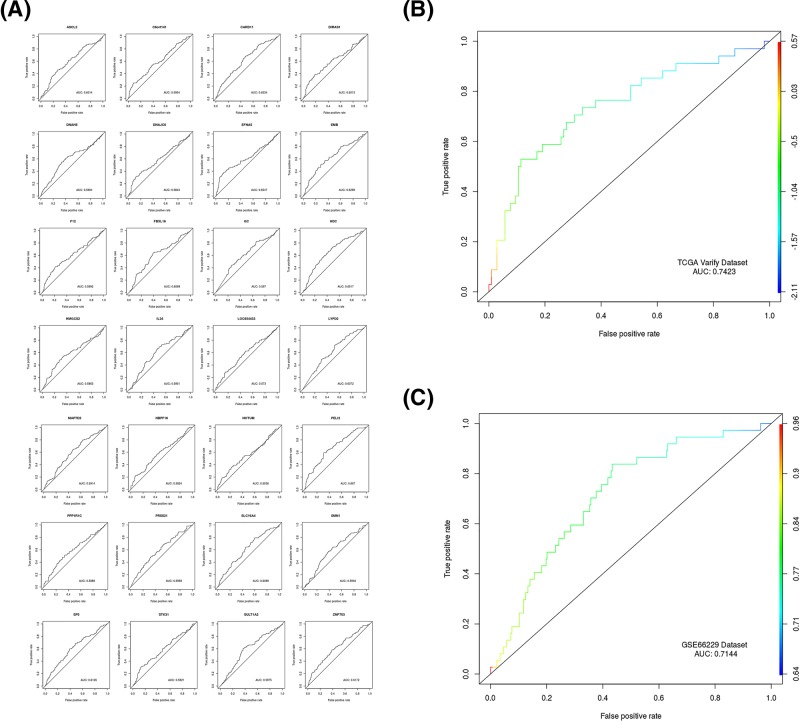
Evaluation of the prognostic performance for 28-gene signature (**A**) The accuracy of each DEG in the diagnosis of lymph node metastasis in GC. (**B**) The accuracy of the 28-gene signature in predicting the lymphatic metastasis of GC. (**C**) GSE66229 dataset was used to validate the accuracy of the 28-gene signature.

First, the AUCs of 28 DEGs in the testing set were calculated separately. The AUC of PELI2 was 0.667, while the AUC of the other 27 DEGs were no larger than 0.652 (Figure 3A). Amongst 28 DEGs, PELI2 had the highest accuracy in predicting lymphatic metastases.

Second, the AUCs of the 28-gene signature in the testing set was 0.7423, which could accurately predict lymphatic metastases in GC tissues (Figure 3B).

Finally, the AUCs of 28-gene signature in the GSE66229 dataset was 0.7144, which further validates the good prognostic performance of the model (Figure 3C). Collectively, the regression model composed of 28-gene signature could specifically, sensitively, and accurately predict lymphatic metastases in GC tissues.

### PELI2 is highly expressed in GC cells

**Figure 4:**
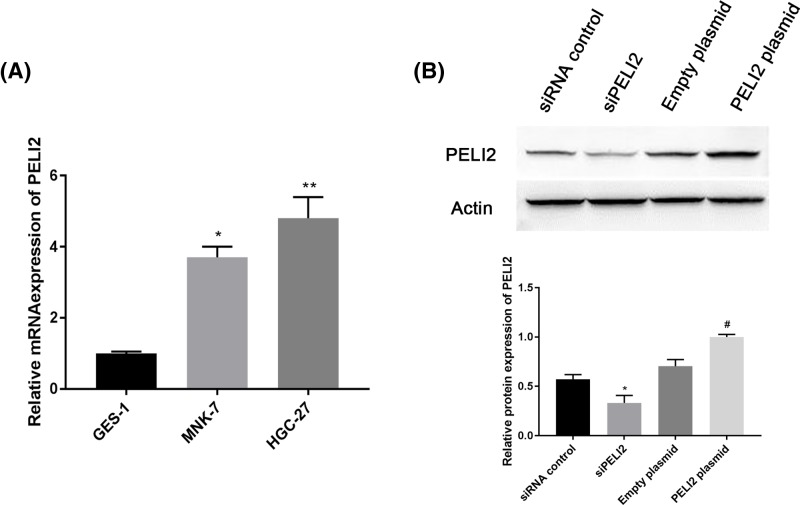
The expression of PELI2 in GC cell lines (**A**) The mRNA expression of PELI2 in normal gastric epithelial cell line GES-1 and GC cell lines (MNK-7 and HGC-27) was determined by qRT-PCR. (**B**) After HGC-27 cells were transfected with siPELI2, siRNA control, PELI2 plasmid or empty plasmid, the protein expression of PELI2 was measured by western blot. *P<0.05 versus control; **P<0.05 versus control; ^#^*P*<0.05 versus empty plasmid.

To further investigate the roles of the 28-gene signature in GC cells, we used a series of biological experiments to determine the effect of PELI2 on GC cells because PELI2 had the most significant differential expression amongst 28 DEGs and the highest accuracy in predicting lymphatic metastases. First, we used qRT-PCR to determine the expression of PELI2 in GC cells. Compared with normal gastric epithelial cell line GES-1, PELI2 was highly expressed in GC cell lines MNK-7 and HGC-27, especially in HGC-27 cell line (Figure 4A). For this reason, HGC-27 cell line was used for further experiments.

After HGC-27 cells were transfected with siPELI2, siRNA control, PELI2 plasmid or empty plasmid, we determined the expression of PELI2 using western blot. siPELI2 effectively decreased the expression of PELI2, and PELI2 plasmid significantly increased PELI2 expression compared with the control group (Figure 4B).

### The effect of PELI2 on proliferation, migration, and invasion of GC cells

**Figure 5:**
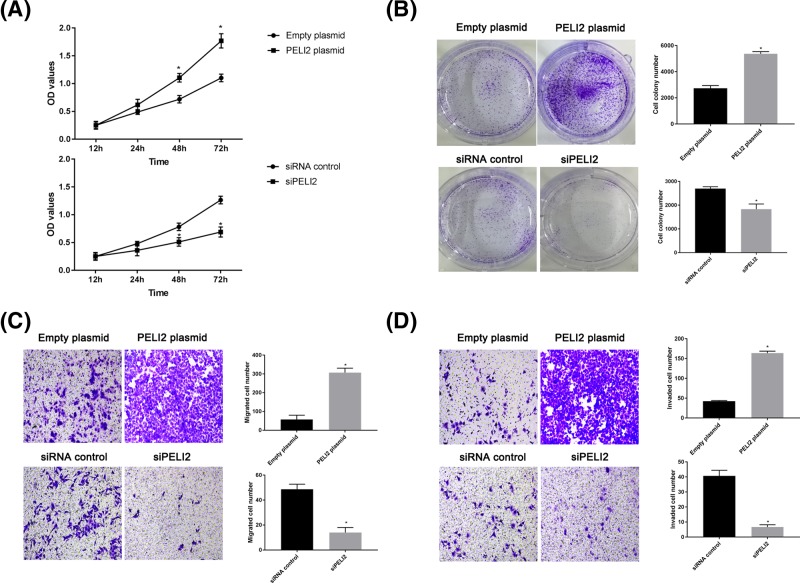
The effect of PELI2 on HGC-27 cells After HGC-27 cells were transfected with siPELI2, siRNA control, PELI2 plasmid or empty plasmid, the viability of HGC cells was determined by CCK-8 (**A**); the colony formation ability of the cells was evaluated by colony formation assay (**B**); the migration (**C**) and invasion (**D**) of the cells were detected by transwell assay. *P<0.05 versus control.

To investigate the role of PELI2 in the proliferation of HGC-27 cell, we used CCK-8 and colony formation assay. CCK-8 showed that PELI2 overexpression clearly enhanced the viability of HGC-27 cells in a time-dependent manner (Figure 5A). Moreover, the number of colonies formed was higher in the PELI2 overexpression group, while the knockdown of PELI2 reduced the colony formation ability of HGC-27 cells, as compared with the control group (Figure 5B). Thus, PELI2 overexpression promoted the proliferation of HGC-27 cells.

Transwell assay was used to evaluate the migration and invasion of GC cells. The number of migrated or invaded cells was increased after HGC-27 cell were transfected with PELI2 plasmid, while the cell number was decreased by the knockdown of PELI2, as compared with the control group (Figure 5C,D). Therefore, the overexpression of PELI2 promoted the migration and invasion of HGC-27 cells.

### The effect of PELI2 on lymphangiogenesis in GC cells

**Figure 6:**
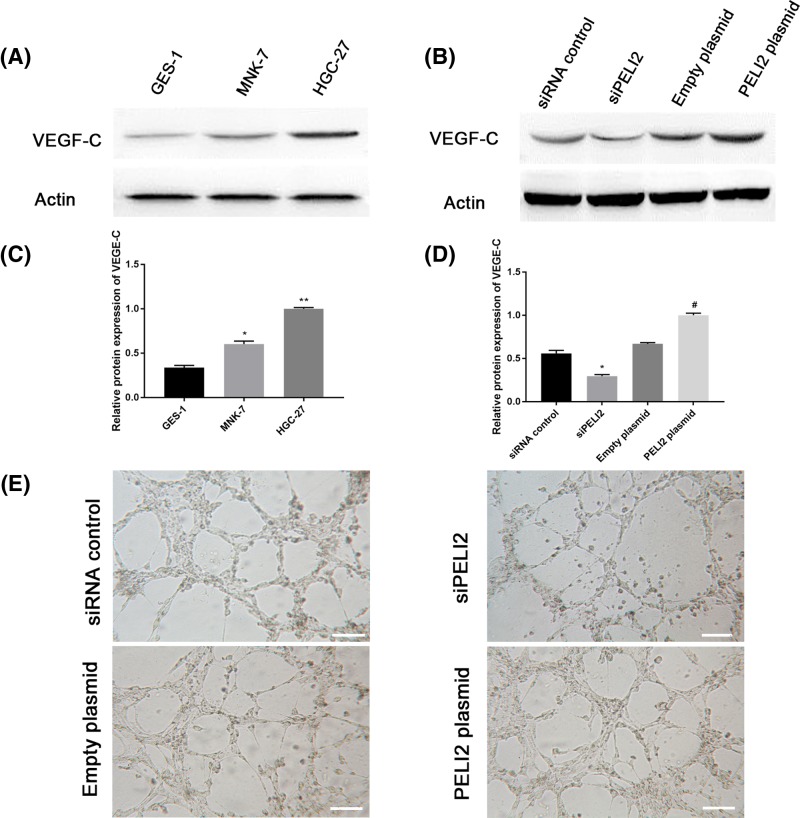
The effect of PELI2 on lymphangiogenesis in GC cells (**A**) The expression of VEGF-C in normal gastric epithelial cell line GES-1 and GC cell lines (MNK-7 and HGC-27) was assessed by western blot. (**B**) Quantitation of VEGF-C expression in GES-1, MNK-7, and HGC-27 cell lines. (**C**) After HGC-27 cells were transfected with siPELI2, siRNA control, PELI2 plasmid or empty plasmid, the expression of VEGF-C in HGC-27 cells was detected by western blot. (**D**) Quantitation of VEGF-C expression in HGC-27 cells. (E) The images of the tube formation assay of HGC-27 cells were recorded using a microscope (scale bar, 50 μm). *P<0.05 versus control; **P<0.05 versus control; ^#^*P*<0.05 versus empty plasmid.

VEGF-C is considered as an important lymphangiogenic growth factor critical for tumor lymphangiogenesis [15]. To investigate the effect of PELI2 on lymphangiogenesis in GC cells, we first assessed the expression of VEGF-C in GC cell lines (MNK-7 and HGC-27) using western blot. VEGF-C expression was greatly up-regulated in MNK-7 and HGC-27 cells compared with the normal gastric epithelial cell line GES-1 (Figure 6A,B). Moreover, the expression of VEGF-C was remarkably enhanced by the PELI2 plasmid transfection, while siPELI2 clearly inhibited VEGF-C expression in HGC-27 cells compared with the control group (Figure 6C,D).

Next, we performed a tube formation assay to further explore the effect of PELI2 on lymphangiogenesis in HGC-27 cells. As compared with the control group, the conditioned medium from HGC-27 cells transfected with PELI2 plasmid significantly promoted the tube formation, whereas the conditioned medium from HGC-27 cells transfected with siPELI2 markedly inhibited the formation of the tubes (Figure 6E). Therefore, PELI2 promoted the lymphangiogenesis in GC cells.

## Discussion

The present study utilized the RNA-Seq data and the elastic net-regularized COX proportional hazard model to identify the 28-gene signature for predicting the lymphatic metastases in GC. Moreover, the 28-gene signature had a better prognostic performance compared with individual DEGs. To further investigate the roles of the 28-gene-signature in GC cells, we selected the gene PELI2 with the best prognostic performance amongst the 28 DEGs for further study. The results revealed that PELI2 is highly expressed in GC and promotes the proliferation, migration, and invasion of GC cells. PELI2 enhanced the expression of VEGF-C and induced the lymphangiogenesis in GC cells.

Although an early diagnosis can improve the prognosis for GC patients, patients tend to be diagnosed at the advanced stage [4]. As the presence of lymphatic metastasis is one of the most important prognostic predictors for GC, early identification of lymphatic metastases is a potential research direction for improving the survival rates [6]. To explore this research direction, we evaluated the RNA-Seq data from TCGA database to obtain a total of 212 DEGs between lymphatic and non-lymphatic metastases in GC tissues. Then, the elastic net-regularized COX proportional hazard model was used to construct a prediction model of lymphatic metastasis. The prediction model consisted of the 28-gene signature, which includes the genes ASCL2, C6orf141, CARD11, DIRAS1, DNAH5, DNAJC6, EFNA5, EMB, F12, FBXL16, GC, HDC, HMGCS2, IL24, LOC654433, LYPD2, MAP7D2, NBPF16, NOTUM, PELI2, PPP1R1C, PRSS21, SLC16A4, SMN1, SP5, STK31, SULT1A2, and ZNF703. Furthermore, the 28-gene signature analyzed by AUC in the testing set had a better prognostic performance than any individual DEG. The good prognostic performance of the 28-gene signature was validated using the GSE66229 dataset. Therefore, the 28-gene signature reported here could be considered as a sensitive predictive tool for lymphatic metastasis in GC.

To further investigate the roles of the 28-gene signature in GC cells, we selected PELI2 for further study, as PELI2 had the most significant differential expression and the highest accuracy in predicting lymphatic metastasis amongst the 28 DEGs. PELI2 encodes pellino-2 protein, a member of Pellino protein family, and plays an important role in cytokine production and innate immune system [16,17]. Pellino family proteins are evolutionary conservative proteins with intrinsic E3 ubiquitin ligase activity. These proteins function as upstream mediators in Toll-like receptor (TLR) pathway, which leads to activation of MAP kinases and transcription factors [18]. Members of the TLR and interleukin-1 receptor (IL-1R) family play important roles in immunity and inflammation. Proteins of TLR and IL-1R pathways initiate common intracellular signaling cascades leading to the activation of NF-kB [19]. Pellino proteins have been suggested to function as evolutionary conserved scaffold proteins in TLR/IL-1R signaling. More recently, it was found that Pellino proteins can catalyze polyubiquitylation of the key TLR signaling molecule IRAK1 (IL-1R associated kinase 1). Kim and coauthors [17] demonstrated that Pellino 2‐mediated IRAK‐1 polyubiquitination plays a critical role for TLR/IL-1R-mediated post-transcriptional control. In addition, PELI2 has been reported to play a role in postmenopausal osteoporosis [20] and hypertension [21].

In the present study, the analysis of the dataset from TCGA showed that PELI2 expression was up-regulated in GC with lymphatic metastasis as compared with GC without lymphatic metastasis. Moreover, the high expression of PELI2 in GC cells was validated by qRT-PCR. Furthermore, PELI2 promoted the proliferation, migration, and invasion of GC cells. In addition, PELI2 enhanced the expression of VEGF-C. VEGF-C is an important lymphangiogenic growth factor, which induces tumor metastasis through promoting cell invasion, lymphangiogenesis, and angiogenesis [15]. In agreement with the previous reports, our study shows that the high expression of VEGF-C induces lymphangiogenesis in GC cells. Therefore, PELI2 could promote the growth and metastasis of GC cells through regulating VEGF-C. To comprehensively understand the mechanism of the identified signature and investigate, the roles of other 27 DEGs in GC, further experimental studies will need to be performed.

In conclusion, with the use of TCGA database and the elastic net-regularized COX proportional hazard model, our study identified a 28-gene signature as a potential prognostic tool for lymphatic metastasis in GC.

## Abbreviations

AUC: area under the curve
CCK: cell counting kit
DEG: differentially expressed gene
FC: fold change
GC: gastric cancer
GEO: Gene Expression Omni-bus database
GES-1: gastric epithelial cell line
HLEC: human lymphatic endothelial cell
ROC: receive operating characteristic
qRT-PCR: quantitative real-time PCR
TCGA: The Cancer Genome Altas
VEGF-C: vascular endothelial growth factor C
